# *Mycobacterium indicus pranii* therapy induces tumor regression in MyD88- and TLR2-dependent manner

**DOI:** 10.1186/s13104-019-4679-0

**Published:** 2019-10-07

**Authors:** Pawan Kumar, Gobardhan Das, Sangeeta Bhaskar

**Affiliations:** 1 PDC-I, National Institute of Immunology, Aruna Asaf Ali Marg, New Delhi, 110067 India; 2 Present Address: Dept. of Preventive Oncology, Dr. B. R. Ambedkar Cancer Hospital, All India Institute of Medical Sciences, Ansari Nagar, New Delhi, 110029 India; 3 Special Centre for Molecular Medicine, Jawaharlal Nehru University, New Delhi, 110067 India

**Keywords:** *Mycobacterium indicus pranii*, Tumor regression, TLR, MyD88, Mouse tumor model

## Abstract

**Objectives:**

*Mycobacterium indicus pranii* (MIP) is an atypical mycobacterium species with potent antitumor efficacy. Macrophages and dendritic cells (DCs) are antigen-presenting cells, playing key roles in the activation of antitumor immunity. We have previously shown the potent activation of macrophages and DCs by MIP, which is mediated by MyD88–TLR2 signaling axis. In the present study, we further examined the role of MyD88 and TLR2 in MIP-mediated tumor regression.

**Results:**

Wild-type and MyD88^−/−^ mice were implanted with B16F10 tumor cells, treated with MIP or phosphate-buffered saline (PBS) and monitored for tumor growth. As expected, MIP therapy led to significant tumor regression in wild-type mice. However, antitumor efficacy of MIP was lost in MyD88^−/−^ animals. Both PBS-treated (control) and MIP-treated MyD88^−/−^ mice developed tumors with comparable volume. Since MyD88 relays TLR engagement signals, we analyzed the antitumor efficacy of MIP in TLR2^−/−^ and TLR4^−/−^ mice. It was observed that MIP therapy reduced tumor burden in wild-type and TLR4^−/−^ mice but not in TLR2^−/−^ mice. Tumor volume in MIP-treated TLR2^−/−^ mice were comparable with those in PBS-treated wild-type animals. These results implicated the MyD88–TLR2 signaling axis in the antitumor efficacy of MIP.

## Introduction

*Mycobacterium indicus pranii* (MIP) is an atypical mycobacterium possessing strong immunomodulatory properties [1]. It was selected from a panel of mycobacteria for evoking cell-mediated immunity against *M. leprae* and was approved for the treatment of leprosy in 1998. Small clinical studies, wherein MIP was evaluated against head and neck cancer, bladder cancer, and lung cancer, also suggested the potent antitumor efficacy of MIP [2, 3]. Mycobacterium species vary widely in their antigenicity and biochemical properties. Recent studies have uniquely placed MIP between slow- and fast-growing mycobacteria [4]. Whole-genome sequencing has revealed the higher levels of putative antigenic molecules in MIP, compared with BCG [1].

We have shown previously that MIP therapy results in significant tumor regression and prolongs the survival of tumor-bearing mice [5]. Immunological studies in these animals revealed that MIP therapy promotes tumor-specific T cell responses and NK cell cytotoxicity [5]. It has been shown that macrophages and dendritic cells (DCs) play key roles in the processing and presentation of tumor antigens and mounting of antitumor immunity. Consistently, MIP was found to activate macrophages and DCs and induce T_H_1 polarization potential in these cells [6, 7]. It was further observed that MIP led to macrophage and DC activation in MyD88- and TLR2-dependent manner [6, 7].

In this study, we explored the role of MyD88 and TLRs in MIP-induced tumor regression. Mice were implanted with B16F10 melanoma cells, treated with MIP and monitored for tumor growth. It was observed that MIP therapy resulted in significant tumor regression in wild-type, but not in MyD88^−/−^ mice. Experiments with TLR knockout mice demonstrated that MIP induced comparable tumor regression in wild-type and TLR4^−/−^ mice. However, antitumor efficacy of MIP was significantly reduced in TLR2^−/−^ mice. These findings shed light on the innate immune mechanisms involved in the antitumor efficacy of MIP.

## Main text

### Materials and methods

**Fig. 1:**
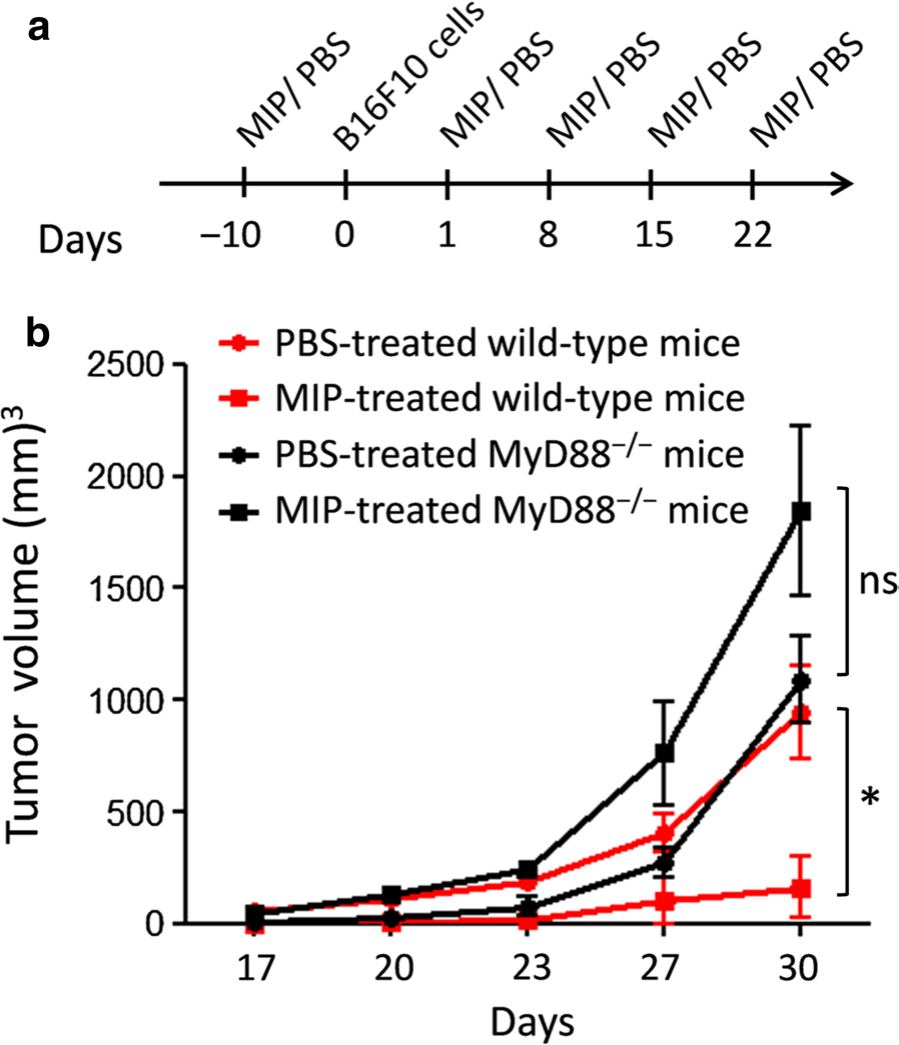
Antitumor efficacy of MIP is lost in MyD88^−/−^ mice. Tumors were implanted in the right flank of wild-type and MyD88^−/−^ mice by injecting B16F10 melanoma cells. Animals were treated with MIP or injected with PBS (control group) using treatment schedule depicted in **a**. Tumor dimensions were measured using Bernier’s caliper and tumor volume were calculated. MIP induced significant tumor regression in wild-type mice, but not in MyD88^−/−^ animals (**b**). Data shown are the mean ± SEM of one of two independent experiments. *p < 0.05; *ns* not significant; n = 6

C57BL/6 and MyD88^−/−^ mice (age 6–8 week) were obtained from the animal house facility of the National Institute of Immunology, New Delhi, India. TLR2^−/−^ and TLR4^−/−^ mice were a kind gift from Dr. Ruslan Medzhitov (Yale University School of Medicine, New Haven, CT). MIP was cultured in Middlebrook 7H9 broth (BD Difco) supplemented with 0.1% Glycerol, 0.05% Tween 80 and 10% albumin-dextrose-catalase enrichment (BD Difco) in a shaking incubator at 37 °C. For MIP therapy, bacilli were harvested, washed and suspended in PBS, and autoclaved for 15 min at 15 lb/in^2^ pressure. Tumors were implanted by s.c. injecting 3.0 × 10^4^ B16F10 cells in the right flank of mice. For MIP therapy, mice were injected peritumorally with 5 × 10^6^ bacilli per 100 μl PBS using dose-schedule shown in Fig. 1a. Control animals were injected with 100 μl PBS using the same schedule. To monitor tumor growth, tumor dimensions were measured with the help of Bernier’s caliper. Tumor volume were calculated using the formula V = 0.5 × L × W × W, where V is tumor volume, L is longer dimension and W is the shorter dimension of tumor. Animals were euthanized after experiments were over by CO_2_ asphyxiation method. Data were analyzed by one way ANOVA (with Tukey’s multiple comparison test applied post-analysis) using GraphPad Prism 5 software.

### Results

Since macrophages and DCs play a key role in mounting of antitumor immunity and are activated by MIP in a MyD88-dependent manner, we asked whether MyD88 also plays a role in the antitumor efficacy of MIP. For this, wild-type and MyD88^−/−^ mice were implanted with B16F10 melanoma cells, treated with MIP and monitored for tumor growth. It was observed that MIP treatment resulted in significant tumor regression in wild-type mice, compared with the PBS-treated control animals (Fig. 1b). However, the antitumor efficacy of MIP was drastically reduced in MyD88^−/−^ mice. MIP-treated MyD88^−/−^ mice and control animals developed tumors with comparable volume (Fig. 1b). These results showed the key role of MyD88-dependent signaling in MIP-induced tumor regression.

**Fig. 2:**
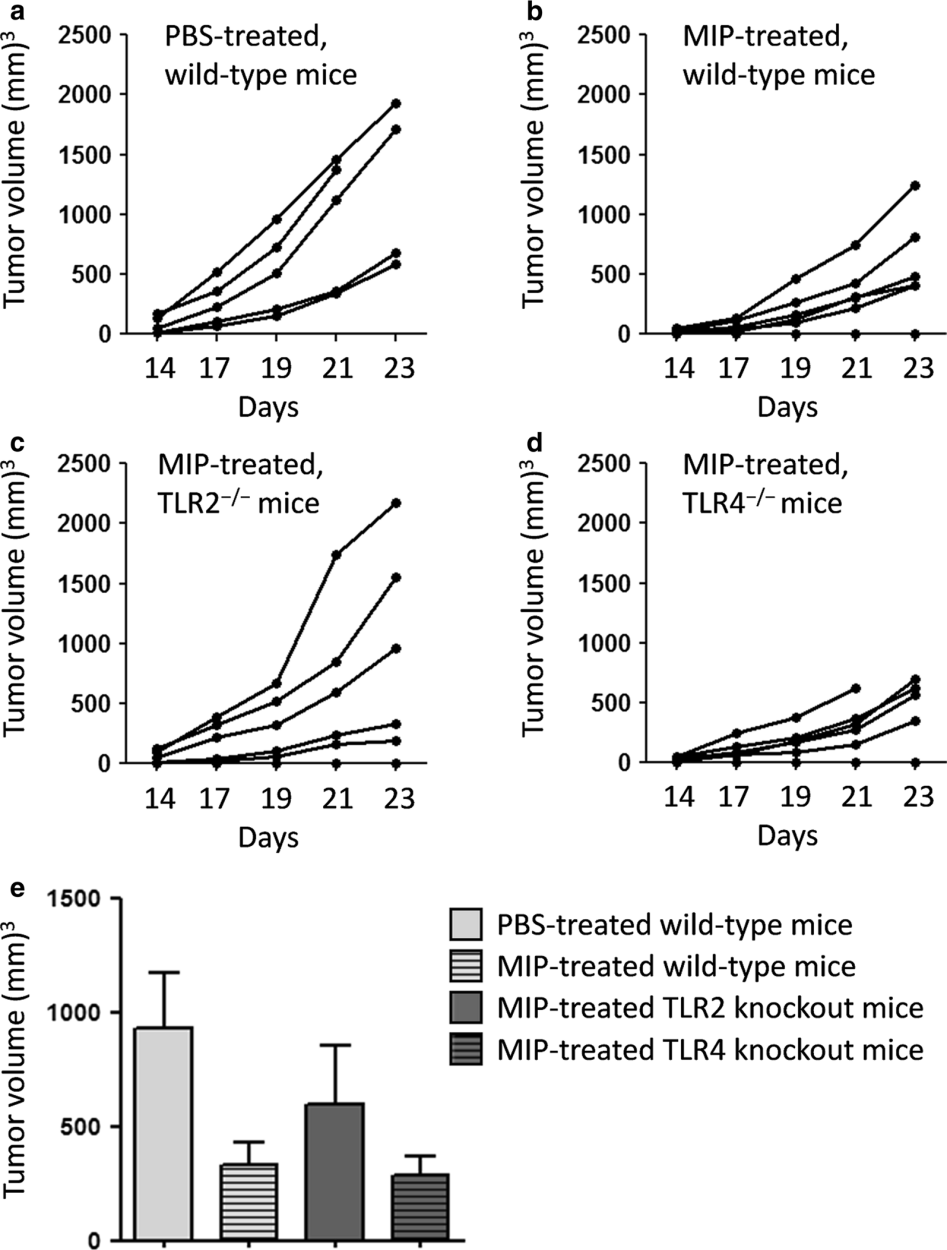
TLR2 plays a key role in the antitumor efficacy of MIP. Wild-type and TLR knockout mice were implanted with B16F10 tumor cells in their right flank, treated with MIP and monitored for tumor growth. Lower tumor volume were observed in MIP-treated wild-type mice, compared with PBS-treated control animals (**a**, **b**). MIP-treated TLR2^−/−^ mice developed large size tumors, as developed by PBS-treated wild-type animals (**c**). MIP-treated TLR4^−/−^ mice developed tumors comparable with those in MIP-treated wild-type animals (**d**). Each curve represents tumor volume in an individual animal. Mean ± SEM of tumor volume in different animal groups on day 21 are shown in the form of bar graph (**e**). Representative data of the one of two independent experiments are shown

In the next experiments, we examined the role of TLRs in MIP-induced tumor regression. TLRs are the evolutionarily conserved trans-membrane pattern recognition receptors employing MyD88 as an adapter protein. Wild-type and TLR^−/−^ mice were implanted with B16F10 tumor cells, treated with MIP and monitored for tumor growth. It was observed that MIP therapy led to significant tumor regression in wild-type and TLR4^−/−^ mice, compared with PBS-treated control animals (Fig. 2a–e). However, the antitumor efficacy of MIP was significantly reduced in TLR2^−/−^ mice (Fig. 2c). Tumor volume in MIP-treated TLR2^−/−^ mice were comparable with those in PBS-treated control animals. Taken together, these results demonstrated the critical role of MyD88–TLR2 signaling axis in the antitumor efficacy of MIP.

### Discussion

The antitumor efficacy of MIP has been demonstrated by both human and animal studies. However, the underlying immune mechanism of MIP-mediated tumor regression remains poorly understood. Here, we explored the roles of MyD88 and TLRs in the antitumor efficacy of MIP for the first time. MyD88 is an adapter protein involved in relaying TLR engagement signals across the plasma membrane [8]. We observed that tumor regression efficacy of MIP was drastically reduced in MyD88^−/−^ mice. Tumors in MIP-treated MyD88^−/−^ mice were larger in size, compared with other groups. Macrophages and DCs are instrumental in mounting of antitumor immunity. Consistent with the above findings, MIP has been shown to activate these cells in a MyD88-dependent manner [6, 7]. *Mycobacterium bovis* BCG is also a potent inducer of antitumor immunity and is used for the treatment of superficial bladder cancer [9]. Similar to our results, Akazawa et al. have shown that the antitumor efficacy of BCG cell-wall skeleton vanishes in MyD88^−/−^ animals [10]. Interestingly, we observed moderately increased tumor volume in MIP-treated MyD88^−/−^ mice, compared with untreated mice. A plausible explanation for this could be drawn from our previous study, wherein we have shown that BCG-stimulated MyD88-deficient DCs secrete substantial amount of inflammatory cytokines but are inefficient in IL-10 secretion [11]. A similar overly inflammatory environment in MIP-treated MyD88^−/−^ mice could lead to increased tumor cell growth, resulting in larger tumor volume.

Next, we examined the involvement of TLRs in MIP-mediated tumor regression. Mycobacteria are known to engage TLR2, TLR4 and TLR9 [12]. We observed that MIP induced significant tumor regression in TLR4^−/−^ mice, but its antitumor efficacy was substantially reduced in TLR2^−/−^ mice. These findings demonstrated that TLR2, but not TLR4 plays a key role in MIP-induced tumor regression. In keeping with these findings, immunostimulatory properties of MIP have been shown to be lost in TLR2-deficient macrophages and DCs [6, 7]. Characterization of TLR2 signaling by heat-killed MIP has shown that TLR2/TLR1 heterodimers play a predominant role in the recognition of the bacilli [7]. Similar to MIP, a synthetic lipoprotein with TLR2/TLR1 engaging property has also been shown to induce tumor regression in the mouse model [13]. Lipoprotein treatment was found to enhance cytotoxic T cell responses and reduce immunosuppressive functions of FoxP3^+^ regulatory T cells [13]. Interestingly, we had also observed higher CTL and NK cell cytotoxicity and reduction of FoxP3^+^ regulatory T cell levels in tumor mass and draining lymph nodes of MIP-treated animals [5].

This study builds upon our previous observations and demonstrates the importance of TLR2 and MyD88 in MIP-mediated tumor regression. Since TLR–MyD88 signaling axis is a key component of innate immunity, these findings implicate the innate immune system in the antitumor efficacy of MIP.

## Limitation

In the present manuscript, we have explored the role of MyD88–TLR signaling axis in the antitumor efficacy of MIP using mouse tumor model. Therefore, these results are specifically valid for the mouse model only.

## Data Availability

Data is available upon reasonable request.

## Abbreviations

MIP: *Mycobacterium indicus pranii*
BCG: Bacillus Calmette–Guerin
TLR: toll-like receptor
PBS: phosphate-buffered saline
DC: dendritic cell
CTL: cytotoxic T lymphocyte
